# A Brief Overview and Update of Regenerative Medicine for the Treatment of Musculoskeletal Conditions

**DOI:** 10.1007/s12178-026-10030-1

**Published:** 2026-05-18

**Authors:** Matthew Negaard, Michael Kelleher

**Affiliations:** 1 Sports Medicine Physician, Forte Sports Medicine and Orthopedics, 10767 Illinois St Suite 3000B, Carmel, IN 46032 USA; 2 Adjunct Clinical Assistant Professor of Emergency Medicine, Indiana University School of Medicine, Indianapolis, IN USA; 3 Adjunct Clinical Assistant Professor of Emergency Medicine, University of Iowa Hospitals and Clinics, Iowa City, IA USA; 4 Emergency Medicine Physician, University of Arizona , Tucson, AZ USA

## Introduction

There is an increasing burden of musculoskeletal disease within the United States and worldwide. Globally, there is an estimated 240 million individuals that have symptomatic osteoarthritis (OA), including 10% of men and 18% of women age 60 and older, with an annual incidence of symptomatic hip and knee OA increasing by 9.3% and 8.2% respectively since 1990 [1]. Within this large aging patient population there is an increasing incidence of complex medical comorbidities that necessitate the development of alternative treatment modalities for various musculoskeletal conditions.

Additionally, there has been advancement in options to treat other chronic conditions such as tendinopathies as well as acute muscle and tendon injuries. To meet the need of the increasing burden of musculoskeletal pathology, numerous regenerative medicine treatments and procedural techniques have been gaining evidence-based support and widespread adoption for use in the outpatient setting. This review provides a brief overview of the current literature and clinical application for the use of platelet-rich plasma (PRP), platelet poor plasma (PPP), alpha-2-macroglobulin (A2M), mesenchymal signaling cells (MSCs) for the treatment of common musculoskeletal conditions.

## Platelet Rich Plasma (PRP)

### Definition, Processing, and Mechanism of Action

PRP is a biological product defined as a portion of the plasma fraction of autologous blood with a platelet concentration above the baseline found in serum blood [1, 2]. PRP is thought to exert its effects through the activation of endogenous cellular pathways that stimulate growth factors, singling cells, cyto- and chemokines, interleukins (ILs), and various cell types; all of which play a role in stimulating and enhancing tissue repair processes [2]. PRP is obtained by venipuncture, followed by centrifugation which allows for the density-dependent separation of blood components, typically into 3 distinct layers. These include supernatant which is PPP, the buffy coat often considered PRP, and infranant consisting of the red blood cell layer (RBCs). In recent years we have seen a significant increase in the literature describing the preparation of PRP; however, no standardized protocol currently exists to optimize its quantity or application [3–6]. 

***Platelet Rich Plasma for Joints***.

#### Knee Joint

PRP is commonly used in the treatment of osteoarthritis (OA), with the knee joint having a substantial body of literature [7, 8]. PRP has been compared to many other conservative injection options for knee OA including hyaluronic acid (HA), corticosteroid injection (CSI), bone marrow aspirate concentrate (BMAC), MFAT and placebo [9, 10].

In 2021, a meta-analysis of level 1 studies comparing PRP and HA for knee OA was performed and concluded that PRP demonstrated a statistically significant improvement in patient reported clinical outcomes compared to HA [7]. This included superior performances in the Western Ontario and McMasters Universities (WOMAC) and Visual Analog Scale (VAS) scales. At 11-month follow-up, 44.7% of PRP patient’s reported significant improvement in WOMAC scores as opposed to 12.6% in the HA group [7]. In 2023, a similar group compared not only PRP and HA but also BMAC for knee OA and found similar results in that PRP and BMAC had improved clinical outcomes with a mean follow up time for 14 months however there was no difference between PRP and BMAC [11]. A similar review paper in 2023 compared the use of CSI, PRP, HA and a combination of PRP + HA injections for knee osteoarthritis [12]. Patient reported outcomes at 3, 6, and 12 months indicated superior clinical outcomes in the PRP and PRP + HA compared to CSI, HA and placebo [12].

#### Hip Joint

PRP has also been used as a conservative injection option for hip OA. However, clinical efficacy is less clear than when compared to knee OA. In 2021, a pilot study looked at 33 intraarticular hip injections comparing PRP vs. HA [13]. Their study demonstrated statistically significant improvement in clinical outcomes at 6 months in favor of PRP compared to HA [13]. Additionally, at 2-year follow up 50% of the HA intervention group had underwent total hip arthroplasty compared to nearly 16% of the PRP group [13]. However, a 2023 meta-analysis including seven randomized controlled trials (RCTs) comparing 1-year clinical outcomes of PRP and HA found no statistically significant difference [14].

#### Other Joints

As seen above in this brief review, the most robust evidence for PRP comes from its use in the treatment of knee OA. However, studies demonstrate its efficacy and use in the treatment of other intraarticular pathology such as glenohumeral OA and carpometacarpal OA. However, a detailed review of PRP for these pathologies is beyond the capacity of this paper.

### Platelet Rich Plasma for Tendon

#### Common Extensor Tendinopathy

PRP has been shown to have superior long term clinical outcomes for patients with common extensor tendinopathy (CET) when compared to corticosteroid injection (CSI) [15]. While CSI may provide superior clinical improvement in the first 2 months after initial injection when compared to PRP, outcomes beyond 6 months significantly favor PRP over CSI [15]. Pintaric et al. utilized computer-aided tendon texture analysis (quantified using gray level run length matrix method (GLRLM) following the injection of PRP in patients with common extensor tendinopathy. The aim was to assess for subtle changes in tissue composition that could be correlated with symptomatic improvement post-injection. In this study, all patients reported symptomatic improvement after PRP injection when assessed using the patient-rated tennis elbow evaluation (PRTEE) questionnaire. Furthermore, of the 8 objective GLRLM features that were used to quantify tissue texture, nearly all demonstrated a statistically significant improvement at 3 months following PRP treatment [16]. Therefore, it appears that PRP may result in long term clinical improvement for CET at 3 to 6 months post-injection.

#### Gluteal Tendinopathy

Several studies have examined the use of PRP for treating gluteal tendinopathy involving the gluteus medius and minimus [27]. Fitzpatrick et al. conducted a double-blind RCT with a two-year follow-up in patients with chronic gluteal tendinopathy. In this study, 80 patients were randomized (1:1) to receive an injection of either LR-PRP or corticosteroid into the gluteus medius or minimus tendons, or the associated bursa. The PRP group demonstrated greater pain improvement compared with the corticosteroid injection (CSI) group [17]. Moreover, the PRP group maintained statistically significant improvement at two years, whereas the CSI group did not show significant clinical improvement beyond 24 weeks [17]. Lee et al. evaluated the efficacy of intratendinous PRP injections for chronic gluteus medius tendinopathy [18]. Patients were assessed pre- and post-injection using the modified Harris Hip Score (mHHS), Hip Outcome Score–Activities of Daily Living subscale (HOS-ADL), Hip Outcome Score–Sport-Specific subscale (HOS-Sport), and the International Hip Outcome Tool–33 (iHOT-33). At a mean follow-up of 19.7 months, statistically significant improvements were observed across all mean clinical scores [18]. These findings provide early evidence supporting the sustained clinical benefits of PRP compared with CSI in the treatment of tendinopathy.

#### Rotator Cuff Tendinopathy

A 2020 systematic review and meta-analysis by Lin et al. included five studies evaluating PRP versus comparators (exercise programs, saline, or dry needling) in patients with clinically or imaging-diagnosed rotator cuff (RC) tendinopathy [19]. Injection sites included the subacromial bursa, intratendinous lesions, and intra-articular spaces; four studies reported repeat injections ranging from 2 to 4 repeat injections [19]. The PRP group demonstrated significantly greater pain reduction, as measured by VAS scores at 24 weeks, compared with controls, but no significant difference in functional outcomes. In a prospective double-blind RCT, Garcia et al. included 84 patients who received either PRP or CSI for chronic RC tendinopathy [20]. Both groups underwent ultrasound-guided injections into the supraspinatus tendon and subacromial space. The PRP group demonstrated statistically significant improvements in clinical and functional outcomes compared with the corticosteroid group at both 6- and 12-month follow-ups [20].

#### Patellar Tendinopathy

In a 2022 review by Masiello et al., which included three studies evaluating the use of PRP in patellar tendinopathy (PT), there was a trend toward improved VAS scores at six and twelve months of follow-up [23]. However these improvements were not significantly different when compared to controls [21]. The Victorian Institute of Sports Assessment–Patella (VISA-P) score was used as a measure of severity of decreased function in tendinopathy, and no significant difference was found amongst PRP versus control groups [23]. Another study by Le et al. looked at the treatment of chronic refractory PT using PRP and included two RCTs. The first study by Dragoo et al. found a statistically significant improvement in short-term symptoms as assessed by VISA-P at 12 weeks, however no difference when assessed at 26 weeks [22]. The second study performed by Vetrano et al. compared the use of PRP with ECSWT and found a statistically significant improvement, as measured by VISA-P and VAS, over ECSWT at 6-month and 12-month follow-up, and as measured by Blazina scale score at 12-month follow-up [22]. There was no difference found between the PRP and ECSWT groups at the 12-week follow-up [22]. Overall, PRP has shown a trend toward improved pain and functional scores at long term follow-up however more studies are needed to establish firm clinical significance for its treatment of PT.

### PRP Factors Contributing to Patient Outcomes

Over the years, several factors within platelet rich plasma and how it is processed and delivered have been investigated to determine their effect on clinical outcomes. Leukocyte classification, grade of osteoarthritis and platelet dose administered have all been factors evaluated predominately in the treatment of knee osteoarthritis.

#### Leukocyte-Rich vs. Leukocyte-Poor PRP

PRP can be processed into different compositions resulting in leukocyte-rich PRP (LR-PRP) or leukocyte-poor PRP(LP-PRP) [17]. Historically, LP-PRP has been favored for intraarticular pathology with the theory being a higher leukocyte concentration leads to a more robust initial inflammatory response resulting in more painful side effects [9]. However, the published literature demonstrates mixed findings regarding this theory [9]. A 2022, meta-analysis evaluating 23 studies with a mean follow up of nearly 10 months comparing LR-PRP to LP-PRP did not show any statistical difference in clinical outcome or adverse events [23]. A RCT performed in 2022 compared the use of LR-PRP and LP-PRP for the treatment of symptomatic knee OA [16]. Their study included 192 patients and demonstrated no significant difference in clinical outcomes or adverse events between the groups at 12 months. That being said, 12.2% of the LR-PRP group reported mild adverse events most commonly described as knee pain and swelling compared to only 4.7% of the LP-PRP group [24]. A subsequent RCT in 2024 comparing LR-PRP and LP-PRP in knee OA showed no difference in clinical outcomes or adverse events up to 12 month post-injection [25] .

#### Grade of OA

Few studies have evaluated PRP’s effectiveness based on the severity of osteoarthritis and the data is mixed [9]. A 2023 meta-analysis included 31 studies that evaluated the use of PRP in patients with early-moderate and severe OA [19]. They found a statistically significant improvement in function and pain scores for patient receiving PRP intervention regardless of the severity of the osteoarthritis [26].

#### Platelet Dose

In recent years, platelet dose has been investigated as a potential source of the heterogeneity observed in studies on the efficacy of PRP. A 2024 systematic review by Berrigan et al. demonstrates that platelet dose may influence clinical outcomes in knee OA, with a therapeutic goal of greater than 10 billion platelets [6]. In this study, they found 14 studies that all achieved positive patient outcomes at 6 and 12-month post-injection when treated with a cumulative dose of > 10 billion platelets following a series of multiple injections [6]. Similarly, Patel et al. found a statistically significant dose-dependent improvement in pain and function at 6 months in patients who were treated with a platelet dosage of 5.65 billion as compared to patients who were treated with a dose of 2.9 billion [27]. Another 2024 systematic review echoed these findings, demonstrating positive outcomes in patients treated with a mean platelet dose of 5.5 billion, whereas those without significant improvements averaged 2.3 billion [28].

Platelet dosing for joints other than the knee have demonstrated mixed results. In patients with hip OA, studies have demonstrated clinical efficacy with PRP dose ranges from less than 5 billion to over 10 billion [6]. However, in the studies included in the review by Patel et al., treatment efficacy only surpassed Hyaluronic acid (HA) when platelet doses exceeded 10 billion [6]. Similar findings were seen for glenohumeral and carpometacarpal OA, with significant improvement in patient reported outcomes seen with a platelet dose of at least 5 billion [6].

#### Platelet Dosing for Tendons

The effect of platelet dose for tendinopathy has been investigated however the optimal therapeutic does remain unclear. There are fewer studies quantifying platelet doses for specific tendinopathies, and unlike intraarticular/joint pathology, the pathogenesis of tendinopathy may complicate the efficacy of PRP making it difficult to discern ideal dosing [6, 29]. In a 2024 systematic review by Averell et al., 20 studies demonstrated a reduction in pain at 6 months after a PRP injection [30]. However, the review failed to demonstrate a statistically significant difference in pain reduction in patients that were treated with “high concentration” vs. “low concentration” PRP [30]. In their review, the low-concentration group ( comprising 12 studies) was defined as PRP concentrations under 5x the concentration of whole blood while the high-concentration group (comprising 8 studies) was defined as concentrations above 5x the concentration of whole blood [30]. The ultimate PRP concentration range found amongst studies in the review was 1.99x-8.0x with a mean of 4.49x [30]. Among studies investigating rotator cuff tendinopathy, there was a reported effective dose range from less than 5 billion to nearly 16 billion [6]. Notably, with larger platelet doses, injections were reported to be spaced out over multiple injections, however there are limited studies comparing the efficacy of single injection vs. multiple injection PRP protocols PRP [6]. While efficacy has clearly been established, the optimal dose for tendinopathy still remains to be seen.

### Platelet Rich Plasma for Ligaments

#### Ulnar Collateral Ligament Injury

Injuries to the ulnar collateral ligament (UCL) are most commonly seen in overhead throwing athletes, particularly baseball pitchers, due to repetitive valgus stress on the elbow. In their cohort study, Mills et al. included 50 throwing athletes with MRI-confirmed UCL tears that underwent injection of PRP [34]. The degree of injury included type I (edema in UCL only; low-grade, partial tear), type II (partial tear of UCL; no extravasation of fluid on arthrogram; high-grade, partial tear), type III (complete, full-thickness tear of the UCL with extravasation of fluid on arthrogram), and type IV (complete tears with additional injury outside the UCL) [31]. Participants received PRP injections directly into the UCL combined with a structured rehabilitation program and were followed for two years after their final injection [31]. 10 of the 50 participants received more than 1 injection with 7 of those participants undergoing reconstructive UCL surgery, despite receiving multiple injections. Overall, 59.5% of participants with type I, II, or III injury returned to their pre-injury level of play following PRP and rehabilitation without requiring surgery. However, only one of eight athletes with type IV injuries returned to their preinjury level of play following the PRP and rehabilitation protocol, with the remainder requiring surgical intervention or retirement [31].

Furthermore, a 2019 review by Apostolakos et al. included a study where 544 professional baseball players underwent non-operative management of UCL injury with or without the addition of PRP [32]. In this study, 133 patients received PRP prior to initiating their nonoperative treatment plan and experienced a significantly longer delay in return to play (RTP) and throwing when compared to the non-PRP group. Ultimately, they found that PRP did not improve RTP outcomes or ligament survivorship in players treated non-operatively [32]. Overall, while the use of PRP has demonstrated some efficacy in mild to moderate UCL injury, additional large studies are required and the gold standard for RTP in patients with severe UCL Injury remains surgical reconstruction.

#### Plantar Fasciitis

Plantar fasciitis (PF) is a common degenerative condition caused by repetitive microtrauma at the fascial insertion [33]. Conservative measures such as rest, analgesics, and physical therapy (PT) remain first-line treatments; however, PRP has been increasingly studied for refractory cases. In a retrospective study, Mannan et al. evaluated 140 patients who received a single PRP injection after experiencing symptoms for more than one month that were unresponsive to conservative management [36]. At 3-month follow-up, 93.5% of patients experienced significant clinical improvement when assessed using VAS and the Foot and Ankle Ability Measure (FAAM) [33]. Similarly, a 2020 RCT by Khurana et al. included 118 patients who received a single injection of either PRP or CSI into the maximally tender aspect of the heel [34]. Outcomes were assessed using the VAS and the American Orthopaedic Foot and Ankle Society (AOFAS) hindfoot score at two weeks, four weeks, three months, and six months. Patients treated with PRP demonstrated statistically significant improvements in both scores compared with those receiving CSI.

Another RCT by Hadad et al. included 110 patients that compared the use of PRP to extracorporeal shockwave therapy (ESWT) in the treatment of PF [35]. The PRP group (*n* = 55) received a single injection into the plantar fascia, whereas the ESWT group (*n* = 55) underwent one treatment per week for three consecutive weeks [38]. Patients were assessed with VAS at 2, 4, 8-, 12-, 16-, and 24-weeks post-intervention. The PRP group demonstrated a statistically significant improvement in VAS scores at 24 weeks compared with the ESWT group [35]. Additionally, a 2025 systematic review and meta-analysis by Rayo-Martín et al. included ten RCTs comparing PRP with CSI in patients with PF. At mid-term follow-up (six months), the PRP group demonstrated significantly lower VAS scores than the CSI group [36].

#### Ankle Sprains

Ankle sprains are one of the most common musculoskeletal injuries in athletes and non-athletes, with the Anterior Talofibular Ligament (ATFL) most commonly injured. Zhang et al. investigated the use of PRP in 83 patients with clinically confirmed grade II lateral ankle sprains (LAS) [37]. Patients were divided into three groups based on injection protocol: no injection (control), a single injection within 48 h after injury (one-injection group), or two injections—one within 48 h and another four weeks later (two-injection group) [37]. All participants were immobilized in a short plaster cast in neutral position for two weeks, followed by an identical rehabilitation program. Pain and function were assessed using VAS and AOFAS scores at two, six, eight, twenty-four, and forty-eight weeks of follow-up. Both PRP groups demonstrated significant improvement compared with controls, with the two-injection group showing statistically superior pain reduction at eight weeks [37]. Pain scores across the three groups were not significantly different at 24 and 48 weeks [37]. Furthermore, MRI was used to assess ATFL quality by calculating the signal intensity and found that all groups demonstrated significant improvement in ATFL quality at 8, 24, and 48 weeks with the two-injection group demonstrating the greatest improvement [37].

When assessing patients with chronic lateral ankle instability (CLAI), Medina-Porqueres et al. injected three rounds of PRP at 7-day intervals both intraarticularly and into the talofibular ligaments of 47 patients [38]. They found statistically significant improvements in functional outcomes as measured by the Cumberland Ankle Instability Tool (CAIT) and Karlsson scores at 3 months [38]. Additionally, Blanco-Rivera et al. evaluated 23 patients with first-time grade II LAS who were treated with rigid immobilization for ten days, with or without the addition of PRP [39]. The experimental group received a single PRP injection into the ATFL and demonstrated significantly greater improvement in Foot and Ankle Disability Index (FADI), VAS, and AOFAS scores compared with the immobilization-only group. However, at twenty-four weeks, outcomes were similar between groups [39].

### Platelet Rich Plasma for Nerve Pathology

#### Median Nerve

Carpal tunnel syndrome (CTS) is caused by the median nerve (MN)’s compression as a result of increased pressure in the bone fiber tunnel formed by the wrist, ligaments, and tendons [40]. This leads to significant pain and functional impairment with clinical symptoms including pain, paresthesia, with progression to muscle weakness and atrophy [41]. A systematic review by Davey et al. included 8 prospective studies with 404 patients comparing the injection of PRP into the carpal tunnel versus control for the treatment of CTS [42]. Controls included CSI, splinting, dextrose 5% in water (D5W), and normal saline (NS). 5 studies reported VAS scores at 3-month follow-up, 5 reported Symptom Severity Scores (SSS) from the Boston Carpal Tunnel Questionnaire (BCTQ) at 1- and 3-month follow-up, and 6 reported Functional Status Scale (FSS) outcomes at 3- and 6-month follow-up. All studies demonstrated statistically significant improvements in the PRP groups compared with controls [42].

Another systematic review and network meta-analysis by Lin et al. (2020) compared therapies including PRP, CSI, D5W, NS, and splinting for the treatment of CTS [41]. Ten studies involving 497 patients were included, with the primary outcome being the standardized mean difference (SMD) in SSS and FSS scores before treatment and at approximately three months of follow-up. The pairwise meta-analysis demonstrated that PRP injections were significantly superior to CSI and NS, while no significant difference was observed between PRP and splinting [41]. D5W was also significantly superior to both CSI and NS [44]. However, in the network meta-analysis, no significant differences were found among PRP, splinting, CSI, and D5W [44].

A 2022 prospective cohort study by Gao et al. included 82 patients who underwent carpal tunnel release (CTR) with or without local PRP injection at the time of surgery [43]. They found no statistically significant differences between groups in VAS, SSS, FSS, or grip strength at 1, 3, or 6 months postoperatively, with both groups experiencing similar rates of symptom remission [43]. In contrast, Chen et al. conducted a double-blind RCT evaluating the one-year efficacy of a single perineural PRP injection in 26 patients (52 wrists) with moderate to severe CTS, with assessments performed at 1, 3, 6, and 12 months [47]. The PRP group demonstrated significant improvements in SSS scores at all time points, significant improvement in FSS scores at 6 months, and a significant reduction in median nerve cross-sectional area at 12 months [44].

### Platelet Poor Plasma (PPP)

#### Basic Science, derivation, and mechanism of action

Platelet poor plasma (PPP) is characterized by a volume of plasma containing a lower concentration of platelets than that found in whole blood [45]. PPP has been demonstrated to exert a beneficial effect on muscle tissue regeneration and healing with beneficial effects on myogenesis, that differ distinctly from PRP [46]. By its definition, PPP contains sub-physiologic concentrations of platelets, however contains numerous bioactive molecules that play a significant role in the promotion of myoblast differentiation, which is an important pathway for appropriate muscle repair and regeneration of functional myocytes.

### PPP in the use of Muscle Injury

In a case report by Kasitinon et al., a 20-year-old American football player underwent injection of PPP for a grade 2 rectus femoris tear that had resulted in 4 weeks of recurrent symptoms and fluid re-accumulation [52]. At one-month post-PPP injection, the athlete was asymptomatic with MRI showing a resolving strain [47]. This was the first case report to be published demonstrating the use of PPP for the treatment of acute muscle injury. Subsequently, a 2025 study by Kruse et al. compared the use of PRP with PPP for the treatment of acute thigh muscle injuries [51]. The study included 100 patients who sustained muscle injuries < 4 weeks prior to injection, with 51 patients receiving PRP and 49 patients receiving PPP. All patients were injected into the region of muscle injury with the primary outcome being number of days until return to full, asymptomatic, unrestricted participation in sport [51]. After adjusting for injury grade, they found that patients treated with PPP experienced a significantly faster return to unrestricted participation in sport, returning 22.89 days sooner than those treated with PRP [51]. Furthermore, there was no significant difference in the injury recurrence rate between groups up to 12 months after initial injury [48].

A retrospective cohort study by Kruse et al. investigated the use of PPP for the treatment of acute grade 3 hamstring injuries in 20 collegiate football players [48]. All subjects sustained injuries within 7 days of evaluation and were diagnosed and graded using ultrasonography and a modified version of the British Athletics Muscle Injury Classification (BAMIC) guidelines [48]. The primary outcome was time to return to full unrestricted participation in sport, with results demonstrating a mean return to full-unrestricted participation being 29.4 days post-injection [45]. This is a significant finding given most studies investigating acute muscle injury demonstrate a mean return to play time of 6–50 + weeks [45].

Finally, Raum et al. reported four cases demonstrating the use of platelet-poor plasma (PPP) for the treatment of acute muscle injury in collegiate and professional athletes [49]. The cases included three collegiate baseball players and one professional baseball pitcher with injuries consisting of a grade II latissimus dorsi strain, a high-grade tear involving the external, internal, and transverse obliques, a full-thickness internal oblique tear, and a grade II strain of the long head of the biceps femoris, respectively [49]. For each case, approximately 12–15 mL of PPP concentrate was prepared and injected under ultrasound guidance into the site of injury. All athletes experienced a returned to play (RTP) in timelines shorter than those previously reported in the literature [53]. Although these cases suggest that PPP may be a promising option for acute muscle injury, further studies are needed to determine optimal injury indications, standardized preparation protocols, and ideal injectate volumes.

##  Alpha 2 Macroglobulin

### Basic science, derivation, and mechanism of action

Alpha-2-macroglobulin (A2M) is a large serum protein produced by the liver that binds and inhibits a wide range of pro-inflammatory mediators and active proteases, thereby limiting downstream tissue destruction [50]. Because many intra-articular pathologies are driven by chronic inflammation and protease-mediated degradation, therapies that interrupt these pathways are essential for effective disease modification. Furthermore, A2M has gained popularity among in-season athletes due to its immediate anti-inflammatory effects, in contrast to PRP, which typically induces a transient inflammatory response before therapeutic benefit occurs. In periarticular conditions such as osteoarthritis, zinc-dependent metalloproteinases, elevated pro-inflammatory cytokines, and activation of macrophages and monocytes contribute to immune-mediated tissue breakdown and progressive disease burden [51, 52]. Tumor necrosis factor-alpha (TNF-α), a key pro-inflammatory cytokine, is believed to act as a master regulator within these destructive pathways [52]. A2M binds to TNF-alpha making is inactive therefore reducing its bioavailability and subsequent downstream tissue breakdown effects.

To investigate the clinical efficacy of A2M, Thompson et al. performed a prospective randomized control trial with 75 patients with symptomatic knee OA. Patients were randomized to one of three cohorts receiving intraarticular injections of either PRP, A2M, or CSI [53]. They found similar clinical efficacy amongst cohorts receiving the A2M and corticosteroid injections, with overall improvement In symptom scoring as compared to the cohort receiving PRP [53]. Five formal pain scoring systems were utilized for clinical assessment; however, only the Western Ontario and McMaster Universities Osteoarthritis Index (WOMAC) demonstrated a statistically significant result [53]. This underscores the need for additional clinical studies to better elucidate the efficacy of A2M as a means to treat periarticular pathology such as OA.

## Mesenchymal Signaling Cells

### Basic science derivation and mechanism of action

Mesenchymal signaling cells (MSCs) are theorized to differentiate into specialized tissue types and contribute to the healing and regeneration of damaged or diseased tissue [54]. Adipose-derived MSCs represent one of the most common and easily isolated sources for clinical use [55]. More specifically, micro-fragmented adipose tissue (MFAT) consists of adipose-derived MSCs originating from vascular pericytes and offers a straightforward method for applying adipose tissue in regenerative procedures due to its ease of extraction through shear stress and microfiltration [55]. MFAT preserves the structural integrity and microarchitecture of the harvested tissue while providing a high concentration of cells and abundant growth factors [56].

Bone marrow aspirate concentrate (BMAC) is an autologous regenerative therapy that has potential in the treatment of intraarticular pathology [57]. BMAC is bone marrow-derived formulation that is rich in MSCs, growth factors, cytokines, and both pro-and anti-inflammatory mediators [57]. Similarly to other autologous MSCs-derived treatments, it exhibits regenerative and immunomodulatory properties primarily due to effects mediated by MSCs. This includes the stimulation of extracellular matrix synthesis and suppression of pro-inflammatory pathways. Furthermore, BMAC is thought to influence subchondral bone remodeling- subsequently enhancing the osteochondral interface which is often involved in the pathophysiology of OA [57]. The preparation process involves aspiration of bone marrow—typically from the iliac crest—followed by centrifugation to concentrate cellular components [57].

### BMAC use in Knee OA

Several studies have evaluated the efficacy of BMAC in the treatment of knee osteoarthritis (OA). In a double-blind RCT, Boffa et al. compared intra-articular BMAC with HA over 24 months in 60 patients, with the primary outcome defined as change in the international knee documentation committee (IKDC) subjective score at six months. They found no significant difference in IKDC scores between groups at six months; however, BMAC demonstrated statistically significant improvements in VAS scores at 12 and 24 months compared with HA [58]. Furthermore, a systematic review performed by Han et al. included 4 studies that compared the use of intraarticular injection of BMAC vs. HA. They demonstrated a statistically significant improvement in the Knee injury and Osteoarthritis Outcome Score (KOOS) and VAS scores at 6- and 12-month follow-up [59]. Notably, these improvements did not reach the minimal clinically important difference (MCID) for either outcome measure. Within the same review, three studies comparing BMAC with PRP reported no significant differences in WOMAC or IKDC scores at twenty-four months [59].

Ruane et al. conducted a single-blinded RCT comparing intraarticular BMAC and PRP injections with a single HA injection as the control [60]. The primary outcome was the KOOS score at 3, 6, and 12 months, with secondary outcomes including the Numerical Pain Rating Scale (NPRS) and the Patient-Reported Outcomes Measurement Information System (PROMIS) Global Health Scale (Global Physical Health [GPH] and Global Mental Health [GMH]) at the same intervals [60]. Both intervention groups demonstrated statistically significant improvements in all primary and secondary outcomes at every time point; however, no significant differences were found between the groups [60].

In a placebo-controlled trial, Shapiro et al. evaluated 25 patients with bilateral knee OA who received a combined BMAC and PPP injection in one knee and a saline injection in the contralateral knee [67]. Patients were followed at one week, 3 months, and 6 months with primary outcomes including the Intermittent and Constant Osteoarthritis Pain (ICOAP) questionnaire and VAS scores. Although both knees exhibited significant improvement from baseline at all follow-ups, there were no significant differences between treated and control knees [61].

### MFAT use in Knee OA

Baria et al. performed a RCT comparing the intraarticular injection of MFAT to PRP in 71 patients with symptomatic knee OA [10]. The primary outcome was the KOOS score at 12-month follow-up, and although both groups showed statistically significant improvement, there was no significant difference between them [10]. In a similar 2022 RCT by Baria et al., 58 patients received a single intraarticular injection of either MFAT or PRP and were assessed using the KOOS score at 6 months post-injection [62]. Both groups demonstrated statistically significant improvement in KOOS scores at six months; however, no significant difference was observed between the two treatments [62]. Finally, Park et al. performed a 2025 systematic review and meta-analysis of six RCTs comparing intraarticular PRP with MFAT [63]. Both treatments produced comparable, statistically significant improvements in pain and functional outcomes, including VAS, KOOS, and IKDC scores [63]. However, at six months, MFAT demonstrated a small but statistically significant advantage over PRP across most clinical measures [63].

### MFAT use in Hip OA

When examining the use of MFAT in the treatment of hip OA, Heidari et al. performed a two-year observational study comparing the intraarticular hip injection of MFAT vs. MFAT plus PRP [64]. Both groups demonstrated significant improvements in VAS and Oxford Hip Score (OHS) at one-year post-injection, with no statistically significant difference between them [70]. Furthermore, a 3-year prospective study by Natali et al. included 55 patients with early hip OA, as determined by the OHS, who received intra-articular MFAT injections over a one-year period [65]. At 3-year follow-up, 28 patients reported significant clinical improvement and required no interim treatment with injections and did not undergo surgery [65]. The remaining patients either underwent additional treatment (CSI injection, viscosupplmentation, PRP, additional MFAT) or underwent total hip replacement. The latter patient group did have a higher degree of moderate and severe graded OA at the start of the study [65].

### BMAC use in Hip OA

In a systematic review evaluating the safety and efficacy of BMAC injections for the treatment of hip OA, Chandrashekar et al. included 5 total studies that all demonstrated clinical improvement with reduction of pain and improvement in function at follow-up intervals ranging from 3 to 12 months [66]. However, more data is needed in the form of RCT (all included studies were cohort or case series) with the use of matched controls.

### MSCs use in Tendinopathy

A systematic review by Imam et al. evaluated studies using BMAC to augment surgical procedures, including rotator cuff repair, lower-extremity tendon repair, and chronic extensor tendinopathy such as lateral epicondylitis. In patients undergoing rotator cuff repair, a case series of 14 patients who received BMAC injection into the affected tendon demonstrated significant clinical improvement based on UCLA Shoulder Rating Scale scores at 12-month follow-up. Furthermore, there was only one revision reported at two years [67]. Another study assessed the use of BMAC for augmentation in arthroscopic single-row rotator cuff repair in 45 patients. They demonstrated superior tendon healing rates and improved tendon quality on ultrasound and MRI compared with matched controls [67]. Additionally, a cohort of 115 shoulders treated with BMAC for glenohumeral osteoarthritis, with or without rotator cuff tear, showed significant improvement in both DASH and VAS scores [67].

The review also described favorable outcomes in Achilles tendon rupture treated with open surgical repair combined with BMAC injection, reporting no recurrent ruptures at an average of 30-month follow-up. Furthermore, 92% of patients reported a return to sport by 6 months [67]. For lateral epicondylitis, one study of 26 elbows refractory to non-operative treatment demonstrated faster recovery and functional improvement following arthroscopic debridement with adjunct BMAC injection, achieving 100% improvement in both VAS and Mayo Elbow Performance Scores at 6 months [67]. Similarly, Singh et al. reported significant improvement in Patient-Rated Tennis Elbow Evaluation (PRTEE) scores at 12-week follow-up in 30 patients treated with BMAC [67].

## Conclusion

Regenerative medicine continues to play an expanding role in the management of musculoskeletal conditions. PRP remains the most widely studied modality demonstrating consistent efficacy across joint, tendon, ligament, and select nerve pathologies, with a growing body of literature supporting its efficacy relative to CSI and HA in appropriately selected patients. Furthermore, recent literature has also demonstrated the statistically significant clinical efficacy of PPP, A2M, and MSCs therapy. However, despite encouraging clinical outcomes, heterogeneity in preparation methods, dosing strategies, and outcome measures continue to limit definitive treatment algorithms. With this in mind, future high-quality, standardized trials are necessary to clarify optimal indications, refine preparation/treatment protocols, and better define the role of these therapies within evidence-based musculoskeletal therapies.

## Key References

- Berrigan, W., et al., *The Effect of Platelet Dose on Outcomes after Platelet Rich Plasma Injections for Musculoskeletal Conditions: A Systematic Review and Meta-Analysis.* Curr Rev Musculoskelet Med, 2024. **17**(12): p. 570–588.
  - ◦ One of the landmark reviews highlighting the importance of the absolute platelet dose and clinical outcomes suggesting there might be a minimum therapeutic dose of platelets for musculoskeletal conditions.
- Kruse, R.C. and E. Volfson, *Platelet-Poor Plasma for the Treatment of Acute Hamstring Muscle Injuries in Collegiate Football Athletes: A Cohort Study.* Clinical Journal of Sport Medicine, 2025. **35**(4): p. 529–533.
  - ◦ This was one of the landmark studies evaluating platelet poor plasma for acute muscle injury which may lead to better clinical outcomes comparted to platelet rich plasma.
- Han, J.H., et al., *Bone Marrow Aspirate Concentrate Injections for the Treatment of Knee Osteoarthritis: A Systematic Review of Randomized Controlled Trials.* Orthop J Sports Med, 2024. **12**(12): p. 23259671241296555.
  - ◦ This is a review of the highest level of evidence for BMAC in the treatment of knee osteoarthritis.<div class="NodiCopyInline">This is a review of the highest level of evidence for BMAC in the treatment of knee osteoarthritis.</div>
- Park, Y.B., et al., *Microfragmented Adipose Tissue as an Alternative to Platelet-Rich Plasma for Intra-articular Injection in Knee Osteoarthritis: A Systematic Review and Meta-analysis of Randomized Controlled Trials.* Am J Sports Med, 2025. **53**(14): p. 3554–3564.
  - ◦ This review compared MFAT to PRP for knee osteoarthritis which help aid in clinical application and discussion of options with patients.

## Data Availability

No datasets were generated or analysed during the current study.
